# Preexisting cognitive impairment in patients with acute stroke

**DOI:** 10.3389/fneur.2025.1629461

**Published:** 2025-11-04

**Authors:** Sarah Kassir, Yannick Béjot

**Affiliations:** Université Bourgogne Europe, CHU Dijon Bourgogne, Service de Neurologie, Dijon Stroke Registry, Dijon, France

**Keywords:** stroke, dementia, cognitive impairment, aging, outcome, frailty

## Abstract

Preexisting cognitive impairment is a significant but often overlooked factor in the management and outcome of stroke patients. Patients with prior cognitive impairment suffering a stroke have less access to stroke units, and less administration of acute revascularization therapies, as a possible consequence of limited research on the benefits of these treatments in this specific population, with most data coming from observational studies. Prestroke cognitive impairment is associated with a greater clinical severity at onset, increased complications, and poorer survival and functional outcome, with a reduced access to rehabilitation services, and a greater need for institutionalization. Patients with preexisting cognitive impairment have more prevalent comorbidities and frailty, which contribute to their increased vulnerability to adverse health outcomes. Further research is needed to better understand how these factors may influence clinical outcomes and decision-making in stroke care in patients with neurocognitive disorders. More inclusive clinical trials and standardized assessment strategies to guide optimal care for this vulnerable population are required. This will be crucial in adapting healthcare systems to meet the needs of a growing and aging population.

## 1 Introduction

Neurocognitive disorders are strong contributors of frailty in elderly people. The risk of developing major cognitive impairment, also referred as dementia, increases with age (1), similar to the incidence of stroke that is 100 folds greater in people over 80 years than in young adults (2). In a context of ongoing aging and growing population, the proportion of patients suffering acute stroke who have preexisting cognitive impairment is rising. While numerous studies focused on post-stroke dementia, less attention has been paid to preexisting cognitive disorders in patients with stroke. This article aimed to depict the burden of prestroke cognitive impairment, its consequences on acute management and patients’ outcome, and relationship with other comorbidities and frailty.

## 2 Prevalence of preexisting cognitive impairment in patients with acute stroke

Only a few studies have evaluated the prevalence of preexisting cognitive impairment in patients with acute stroke. Prior dementia was estimated to range between 9 and 14% according to a meta-analysis (3). In Oxford Vascular Study (OXVASC), the prevalence of prestroke dementia increased with clinical severity of the cerebrovascular event, ranging from 5% in transient ischemic attack to 21% in severe major stroke (4). Mild cognitive impairment (MCI) before stroke has been even less investigated. A population-based study reported a prevalence of preexisting MCI of 14%, alongside with a similar proportion of patients with prior dementia (5). Not surprisingly, patients with prestroke cognitive impairment are older than their non-cognitively impaired counterparts (5, 6). Several methodological limitations can explain this lack of data in the literature. Above all, the assessment of cognitive disorders prior to stroke is challenging. This assessment can be conducted using standardized questionnaires, such as the IQCODE (Informant Questionnaire on COgnitive Decline in the Elderly) that has been shown to have a good accuracy for identifying preexisting dementia in patients with stroke (7), but their use remains limited especially when considering population-based settings. To address this limitation, retrospective epidemiological surveys are alternatives frequently used in observational studies. Finally, study setting (hospital- versus population-based recruitment of patients) and demographic characteristics of population strongly influence estimates of preexisting cognitive impairment and account for variations between studies.

Given the high prevalence of cognitive impairment in stroke patients, it has been hypothesized that it may conversely represent a risk factor for acute stroke. A meta-analysis reported 10 out of 12 studies corroborating this hypothesis with a global relative risk of 1.15 (8). However, a publication bias was not excluded. Moreover, establishing causality remains difficult as chronic vascular lesions contribute to vascular dementia, which may finally represent a clinical manifestation of an underlying neurovascular condition.

## 3 Acute stroke management in patients with preexisting cognitive impairment

Stroke units were progressively implemented over the last decades to improve acute management of stroke patients, and a meta-analysis of available literature suggested that admission to such organized inpatient care was associated with reduced mortality and disability as compared with general wards (9). Of note, the apparent benefits were independent of age, sex, stroke type, and clinical severity. However, no subgroup analysis according to preexisting comorbidities, including prior-to-stroke cognitive impairment, was performed, thus leaving uncertainty about the relevance of this type of care for patients with major neurocognitive disorders.

Several studies pointed out that patients with dementia were less likely to be admitted to (10–12) or have a shorter stay in (13) a stroke unit. In addition, they received a poorer quality of care with reduced access to various diagnostic procedures (11, 14, 15), swallowing assessment (11, 13), interdisciplinary functional evaluation (13), or invasive treatments such as endotracheal intubation (16). Treatment differences were also seen in revascularization decisions in patients with acute ischemic stroke, as studies reported that intravenous thrombolysis (10, 11, 17), and mechanical thrombectomy (10, 18) were less commonly administered to patients with preexisting dementia. The influence of prestroke MCI is less documented, a population-based study reported a similar administration of intravenous thrombolysis but lower use of mechanical thrombectomy in patients with prior MCI compared with those with normal cognition (5). Although evidence regarding sex disparities in access to diagnostic procedures and stroke care remains inconsistent in the general stroke population (14, 19–21), data specifically addressing sex differences among patients with prestroke cognitive impairment are limited. Most studies have treated sex as a confounding variable rather than performing dedicated subgroup analyses (14, 21, 22). Therefore, additional research is required to clarify this issue. Furthermore, disparities in stroke care for cognitively impaired patients appear to be a global phenomenon, with consistent reports from North America, Europe, and Asia. Notably, patients with MCI have also been shown to experience disparities in acute stroke care, including a lower rate of intravenous thrombolysis administration (22).

Several reasons could account for the lower administration of acute revascularization therapies including a greater clinical severity at onset (23), delays in treatment times (11, 17), or reluctance of clinicians with regard to the use of such therapies in severely cognitively-impaired patients. Another contributing factor is the scarcity of high-quality evidence on the effect of acute revascularization therapy in patients suffering ischemic stroke and who have preexisting neurocognitive disorders. Hence, most randomized clinical trials excluded individuals with prior disability (24). Consequently, current evidence on the efficacy and safety of intravenous thrombolysis and mechanical thrombectomy in patients with prestroke dementia exclusively come from observational studies. Although these studies suggested no loss of treatment benefit, an increased risk of symptomatic intracerebral hemorrhage was highlighted (25), and results should be interpretated with caution due to obvious selection bias inherent to such methodological approach. Efforts to enroll patients with premorbid neurocognitive disorders in future randomized clinical trials are urgently needed.

Finally, while treatment disparities have been mainly documented in ischemic stroke (for thrombolysis and thrombectomy), observational data suggest that similar patterns of reduced diagnostic and rehabilitative care also affect hemorrhagic stroke patients with pre-existing cognitive impairment.

## 4 Post-stroke outcome in patients with preexisting cognitive impairment

Several observational studies demonstrated that preexisting dementia was associated with a poorer early (5, 6, 26–29) and long-term (10, 28, 30) survival after stroke. Albeit some diverging conclusions (31), the association was found to be independent of confounding factors including age, sex, and stroke type in most studies. To account for this result it has been observed that patients admitted for a stroke and who have preexisting dementia could be at greater risk of acute medical complications including pneumonia, urinary tract infections, and gastrointestinal bleeding (6, 11, 26, 27). In addition, preexisting cognitive impairment was also associated with post-stroke delirium, thus contributing to a greater risk of in-hospital death (32, 33).

Among other hypotheses, it was assumed that the poorer prognosis after stroke in patients with preexisting cognitive impairment might be related to a greater clinical severity at onset. A population-based study demonstrated that patients with preexisting dementia, and to a lesser extent those with MCI, had more severe ischemic stroke, as measured at admission with the National Institute of Health Stroke Scale (NIHSS) score, than their not cognitively-impaired counterparts (23). Interestingly, the association was still observed after adjustment for well-established confounding factors, and was not mediated by preexisting structural visible brain damages on conventional imaging including old vascular lesion, brain atrophy, and leukoaraiosis (34), thus suggesting an impaired brain ischemic tolerance in patients with prior neurocognitive disorders that may involve processes at a cellular or neuronal network scale yet to be elucidated. However, in this study, the excess in case-fatality in patients with MCI or dementia persisted after adjustment on clinical severity (5), which indicated that a more complex interplay between preexisting cognitive impairment and outcome after stroke. Notably, a gradual effect was observed, whereby the association between dementia and either initial stroke severity or mortality was more pronounced than that observed for MCI, thus suggesting that the severity of cognitive impairment may influence post-stroke outcomes (5, 23). Conversely, whether the underlying cause of cognitive impairment may have an impact on post-stroke outcomes has been only poorly investigated. A study concluded that stroke patients with Alzheimer’s disease or mixed dementia had higher post-stroke mortality compared to those with vascular dementia (30). Furthermore, these outcomes may be also shaped by stroke recurrence, which depends on adequate vascular risk factor control and appropriate treatment. A twofold increase in recurrence risk has been reported among patients with prestroke cognitive impairment. This finding should be interpreted in the context of the well-established association between prestroke cognitive status and the development of post-stroke dementia (29, 35).

Stroke-related disability has a great impact on patients with neurocognitive impairment. Hence, several studies showed that patients with acute stroke and prior dementia had a reduced likelihood of returning to a prior living arrangement with a high risk of institutionalization (11, 36–38), thus highlighting consequences in terms of anticipating current and future needs of dedicated facilities. In a multicenter study conducted in England, patients with prestroke neurocognitive disorders received significantly fewer rehabilitation services, particularly physiotherapy (39). A qualitative assessment of clinical parameters influencing medical decisions about rehabilitation highlighted preexisting neurocognitive disorders as a major contributor of decision-making (40). Multiple barriers may contribute to the reluctance of clinicians to transfer patients with cognitive impairment to rehabilitation including inability to understand and participate in exercises, and behavior disturbances that could hinder the effectiveness of care programs. Therefore, some medical decisions may result from a subjective judgment, with clinicians anticipating a low chance of recovery in patients with preexisting cognitive impairment, in the absence of adapted evidence-based guidelines. Indeed, there are conflicting results in the literature regarding the benefit of rehabilitation after stroke in patient with preexisting cognitive impairment. Although some studies suggested that cognitive impairment negatively affect the effectiveness of rehabilitation, other works concluded to similar functional improvement, especially regarding ambulation, in cognitively normal and impaired patients (41, 42). Further research is needed to better define and evaluate cognitive-adapted rehabilitation programs that accommodate patients’ impairment.

## 5 Impact of other comorbidities on post-stroke outcome

Factors associated with poor prognosis following stroke remain a subject of ongoing research, underscoring the need to better understand and optimize care trajectories, particularly in patients experiencing ischemic stroke (43, 44). From a general point of view, comorbidities or dependency prior to stroke have been shown to be associated with a poorer survival (45, 46), and worse functional outcomes after rehabilitation (47). She et al. studied the impact of 16 chronic diseases, grouped together by categories (e.g., cardiopulmonary, hepatogastrointestinal, metabolic-renal, depression), all of which negatively affected functional and cognitive outcomes after a stroke (48). In most studies focusing on this topic, comorbidities were assessed by scores, the most frequently used being the Charlson Comorbidity Index, which was shown to positively correlate with an increased risk of death, particularly with conditions like cancer, renal, or liver disease. Notably, this index identifies dementia as a comorbidity alongside a list of other diseases, with results interpreted as a cumulative effect of each comorbidity, without considering potential interactions between them. Further works are needed to examine specifically the impact of comorbidities on management and outcome of stroke patients with preexisting neurocognitive disorders.

Another consideration is the complex interrelationship between cognitive impairment, frailty, and stroke. Frailty is a well-established risk factor for dementia (49, 50), and patients with neurocognitive disorders are at greater risk of having frailty than their non-cognitively impaired counterparts (51). On the other hand, there is growing evidence supporting a negative impact of frailty on post-stroke outcome. Hence, similarly to neurocognitive disorders, frailty seems to be associated with higher mortality, length of hospital stay, and disability after stroke (52–57). In a prospective study, frail patients had a higher in-hospital mortality, with a greater prevalence of deep venous thrombosis, and frailty emerged as best predictor of death at 12 months after ischemic stroke treated with acute revascularization therapy (56). In terms of recovery, functional improvement was shown to be largely reduced in frail versus robust patients (54). The risk of institutionalization after stroke was also greater in patients with frailty (58). To date, the mechanisms underlying these pejorative correlations remain unclearly explained. In secondary analysis of a cohort study, heterogeneity in trajectories after stroke was identified among frailer patients, and interactions with various variables including comorbidities and poor self-reported health were suggested (59). Additional research will help to clarify the individual contribution of frailty on post-stroke outcome in cognitively impaired patients.

## 6 Discussion

Based on relevant studies on this topic, the main ones of which are summarized in Table 1, this narrative review highlights the growing recognition of preexisting cognitive impairment as a major determinant of stroke trajectories.

**Table 1:** Summary table of selected relevant studies on prestroke cognitive impairment, frailty, and post-stroke outcomes, and research perspectives.

Authors (Year)	Study type	Main findings	Perspectives
Preexisting cognitive impairment and stroke outcomes			
Pendlebury S et al. (2009) (3)	Systematic review and meta-analysis	Estimated prestroke dementia prevalence: 9–14%; Age, medial temporal lobe atrophy, family history of dementia, global cerebral atrophy, previous stroke, leukoaraiosis, diabetes, atrial fibrillation, hypertension were associated to prestroke dementia.	Short global cognitive tests sensitive to mild cognitive impairment would help to determine the relative contributions of, and interactions between, degenerative and vascular processes in the etiology of post-stroke dementia
Pendlebury et al. (2019) (4)	Population-based study (OXVASC study)	Prestroke and post stroke dementia were both associated with the severity of cerebrovascular events. Prevalence of post-event cognitive impairment: ~5% after TIA to ~21% after major stroke.	Improvements in acute stroke care to reduce lesion severity might decrease immediate disability but also have an impact on long-term risk of dementia. Specific treatment (thrombectomy, thrombolysis).
Graber M et al. (2020) (5)	Population-based study (Dijon Stroke Registry)	MCI and dementia before stroke were associated with increased post-stroke mortality and poor functional outcome, after adjustment for stroke severity and vascular risk factors.	Some factors may interfere in the association between the level of cognitive impairment and poor prognosis at short- and mi-term.
Hénon H et al. (2003) (6)	Prospective cohort study	Prestroke dementia increased early and late mortality at 3-years	Causes of death did not differ between cognitively impaired and not impaired patients. Mortality gaps might be partly explained by different therapeutic approaches.
Van Nieuwkerk AC et al. (2021) (7)	Population-based study (OXVASC study)	The IQCODE score was accurate for detecting preexisting dementia in patients with cerebrovascular events.	Further studies may examine accuracy of IQCODE in differentiation of dementia subtypes, also its predictive value for poststroke cognitive decline.
Béjot Y et al. (2023) (10)	Population-based study (Dijon Stroke Registry)	Case fatality rates at 5 years rose with level of prestroke cognitive impairment: 38.1% in patients without cognitive impairment, 65.9% in patients with prior MCI and 86.6% in patients with prior dementia.	Cognitive screening should be incorporated into population-based outcome monitoring.
Saposnik G et al. (2011) (11)	Nationwide study (Canadian Stroke Network)	Patients with preexisting dementia had higher disability at discharge (OR 3.20) and were less likely to be discharged to their prestroke place of residence.	Adapted recommendations are needed regarding patients with cognitive impairments to help medical decision-making. Further studies are needed to explore prognostic factors in this population.
Callisaya ML et al. (2021) (12)	Observational cohort study	Dementia was associated with poorer quality of care (less admission to stroke units, IV thrombolysis, access to physiotherapy or dietitian visit) after stroke, and worse clinical outcomes, indicating care disparities for cognitively impaired patients	Disparities in quality of care regarding cognitive status raise equity issues, highlighting the need to better understand underlying reasons.
Zupanic E et al. (2018) (13)	Cohort from Swedish dementia & stroke registries	Patients with dementia had equal access to stroke unit care compared with non-dementia patients, but duration of stay was shorter. Patients with dementia were less likely to receive diagnostic procedures.	The specific impact of dementia on outcome should be better understood in order not to limit investigations in patients who could benefit from diagnostic tests.
Béjot Y et al. (2015) (14)	Population-based study (Dijon Stroke Registry)	Patients with dementia were less likely to undergo diagnostic procedures after ischemic stroke.	Whether lesser diagnostic explorations could account for the reported excess in recurrent events is a hypothesis.
Subic A et al. (2017) (15)	Observational / registry analyses	Review of management of acute ischemic stroke in patients with dementia pointed differences in acute care and decision-making compared with non-demented patients.	This highlights the need for equitable acute care and standardized protocols for cognitively impaired patients.
Busl KM et al. (2013) (16)	Observational study	Prestroke dementia was associated with poorer outcomes after acute reperfusion therapy in elderly patients.	Supports inclusion of cognitive status in reperfusion eligibility research.
Zupanic E et al. (2017) (17)	Nationwide study (Swedish Registry)	Patients with dementia were less frequently treated with thrombolysis. Outcomes were similar when treated.	Carefully selected patients with prior dementia could still benefit from reperfusion. Highlights the need for prospective validation.
Ganesh A et al. (2022) (25)	Guidelines (AHA Stroke Council)	A better ascertainment of premorbid disability and cognition, and inclusion of their status for reperfusion therapies strategies are suggested.	Provides framework for integrating cognitive status into guidelines and outcome measures.
Liu C et al. (2022) (26)	Multicenter registry (Chinese Stroke Center Alliance)	Worse in-hospital outcomes in patients with prestroke dementia.	Reinforces global relevance of cognitive screening.
Appelros P et al. (2005) (27)	Observational hospital study	Stroke-related death was higher in patients with premorbid cognitive impairment, even after adjustment for contributing factors.	Patients with prestroke cognitive impairment may be highly susceptible to neurovascular damages.
Yu H et al. (2022) (29)	Systematic review and meta-analysis	Both prestroke MCI and dementia were associated with higher mortality after stroke. Patients with cognitive impairments had a twofold risk of stroke recurrence.	Changes in cerebrovascular hemodynamics may be determinants of poor cognitive and functional outcome after stroke.
Zupanic E et al. (2021) (30)	Registry-based cohort	Prestroke dementia was an independent predictor of death. Patients with Alzheimer’s disease or mixed dementia had higher post-stroke mortality compared to those with vascular dementia	Differentiating dementia etiologies might be relevant to predict outcomes in cognitively impaired stroke patients.
Saposnik G et al. (2012) (31)	Propensity matched cohort (Canadian Stroke Network)	There were no difference in survival at either discharge, 30 days, or 1 year after stroke between patients with and without dementia.	Dementia *per se* may not fully account for observed poorer outcomes, which could be partly explained by differences in baseline characteristics of patients such as stroke severity and comorbidities. Pre-existing dementia should not limit access to specialized stroke care.
Pinguet V et al. (2022) (34)	Population-based study (Dijon Stroke Registry)	The association between pre-existing cognitive impairment and clinical severity in patients with ischemic stroke was not fully explained by visible structural lesions on brain imaging	Other mechanisms including impaired brain ischemic tolerance in patients with prestroke cognitive impairment remain to be elucidated
Kwan A et al. (2021) (35)	Prospective cohort study	Cognitive impairment after lacunar stroke independently predicted higher risk of recurrent stroke and death.	Post-stroke cognitive assessment should be integrated into risk stratification for recurrence and long-term management of lacunar stroke survivors.
Béjot Y et al. (2021) (36)	Population-based study (Dijon Stroke Registry)	Prior cognitive impairment predicted worse early outcomes.	Reinforces the need for geriatric assessment in acute stroke management.
Garcia-Ptacek S et al. (2018) (37)	Cohort study	Premorbid dementia and poor mobility strongly predicted unfavorable outcomes.	Functional and cognitive frailty must be considered in post-stroke planning of care.
Pasquini M et al. (2007) (38)	Prospective cohort study	Cognitive impairment increased the risk of institutionalization after stroke.	Underlines the importance of early community and caregiver support interventions.
Longley V et al. (2019) (39)	Cohort study	Patients with prestroke cognitive impairment received significantly less stroke rehabilitation compared to their non cognitively impaired counterparts, even after adjustment for stroke severity and functional status	Suggests potential inequities in rehabilitation allocation and emphasizes the need for systematic assessment and adapted access for patients with prior cognitive impairment.
Longley V et al. (2018) (40)	Qualitative study (semi-structured interview)	Clinicians reported that decisions to continue rehabilitation were influenced by perceived potential for improvement, behavioral issues, and family support	Highlights implicit bias in access to post-stroke rehabilitation for cognitively impaired patients. There is a need for clear guidelines and to support equitable rehabilitation decisions.
Rabadi MH et al. (2008) (42)	Cohort study	Changes in FIM scores were not different in patients with MCI or dementia compared to control group. Cognitively impaired stroke patients still benefited from rehabilitation.	Rehabilitation services should not be restricted to individuals with cognitive impairment, since they demonstrate similar levels of benefit from such programs.
Impact of comorbidities on post-stroke outcome			
Corraini P et al. (2018) (45)	Population-based cohort	Higher comorbidity burden associated with increased mortality after stroke.	Multimorbidity should be integrated into prognostic models and personalized care planning.
Sennfält S et al. (2021) (46)	Observational cohort study	Prestroke dependency was associated with distinct patient characteristics and predicted poorer long-term outcomes.	Prestroke dependency metrics should inform discharge planning and rehabilitation intensity.
Patrick L et al. (2001) (47)	Observational cohort study	Greater medical comorbidity reduced rehabilitation efficiency in older inpatients.	Global geriatric assessment and consideration of comorbidities could optimize rehabilitation programs in comorbid older patients.
She R et al. (2022) (48)	Multicenter cohort study	Specific comorbidity patterns were associated with worse cognitive and physical function after stroke: degenerative-cardiopulmonary, heart- gastrointestinal-psychiatric, and metabolic-kidney diseases	Comorbidity clusters could be targeted for prevention and tailored post-stroke rehabilitation.
Frailty and post-stroke stroke outcome			
Kojima G et al. (2016) (49)	Systematic review & meta-analysis	Frailty predicted higher risk of Alzheimer’s disease, vascular dementia and all-cause dementia.	Screening for frailty could identify populations at risk for cognitive decline and post-stroke complications.
Koria LG et al. (2022) (51)	Systematic review	There was a high prevalence of frailty among people with dementia. Frailty represented 50.8 to 91.8% of patients with dementia among studies conducted in acute care setting. Amount of medication-use was associated with frailty.	Frailty should systematically be documented in patients with cognitive impairments.
Binning L et al. (2025) (52)	Systematic review & meta-analysis	Brain frailty was assessed using imaging markers. Cognitive reserve was assessed using the Cognitive Reserve Index Questionnaire. Cognitive frailty and low cognitive reserve were associated with worse stroke outcomes and recovery.	Brain frailty is common in stroke and is associated with poorer outcomes but the epidemiology of cognitive frailty and reserve in global population need to be elucidated to better understand their impact on prognosis.
Yang F et al. (2022) (54)	Prospective cohort study	Prestroke frailty predicted poorer prognosis and functional outcomes.	Prestroke frailty screening could help to plan proper rehabilitation for patients.
Zhang Q et al. (2023) (55)	Cohort study	Prestroke frailty and related health factors were associated with lower post-stroke functional independence, evaluated by ADL and IADL scales.	Comprehensive assessment of frailty may help to identify those with most significant risk for declining functional capacities after stroke to provide appropriate care.
Pilotto A et al. (2022) (56)	Cohort study	Frailty predicted worse improvement at 24 h, higher in-hospital mortality and poorer survival at 3 and 12 months.	Larger longitudinal studies are needed to evaluate the risk–benefit of reperfusion treatment in the growing elderly frail population.
He H et al. (2024) (57)	Multicenter prospective cohort study	Baseline frailty was associated with post-stroke disability after adjusting for socio-demographic, clinical variables and baseline mRS. Frailty was an independent risk factor for non-selective readmission.	Evaluating the frailty status at admission could help to improve short term outcome after a stroke.
Cui Y et al. (2024) (58)	Prospective study	Patient-reported frailty was associated with higher rates of non-home discharge, and prolonged length of stay.	Frailty assessment may improve post-stroke care pathways and strategies. FRAIL scale can be used at acute stage as a practical screening tool.
Zeng W et al. (2024) (59)	National cohort study	Identified trajectories of physical frailty post-stroke and associations with outcomes.	Track frailty trajectories longitudinally to personalize rehabilitation and community support.

ADL, Activities of Daily Living; CI, Confidence Interval; FIM, Functional Independence Measure; FRAIL scale, Fatigue Resistance Ambulation Illnesses and Loss of weight scale; IAD, Instrumental Activities of Daily Living; MCI, Mild Cognitive Impairment; MMSE, Mini-Mental State Examination.

Current literature pointed out a poorer outcome after stroke in patients with preexisting cognitive impairment, with data suggesting differential clinical management as a contributing factor. Given their complex health status, these patients are particularly vulnerable to early complications after stroke. In a context of growing and aging population, it is critical to further investigate the impact of comorbidities and frailty on post-stroke outcome, especially in patients with neurocognitive disorders, so as to better guide clinicians for treatment decisions, and help policy-makers to better anticipating resources allocation.

A statement from the American Heart Association/American Stroke Association recently pointed out the need for a better ascertainment and measurement of premorbid disability and cognitive impairment in the setting of acute stroke, thus requiring harmonized and validated strategies (25). These strategies include the use of clinical tools such as standardized scores in a more systematic way. There remain challenges in assessing profile of patients that would benefit the most from each strategy of care from the acute stage to post-stroke rehabilitation and follow-up. At the global level, evidence indicates disparities in the prevalence of cognitive disorders across ethnic group (60) and in stroke risk between low- and high-income countries (61, 62), thus emphasizing the importance of ethnically and socioeconomically inclusive research.
